# Contemporary Management of 1–4 Brain Metastases

**DOI:** 10.3389/fonc.2018.00385

**Published:** 2018-09-24

**Authors:** Sarah M. C. Sittenfeld, John H. Suh, Erin S. Murphy, Jennifer S. Yu, Samuel T. Chao

**Affiliations:** Department of Radiation Oncology, Rose Ella Burkhardt Brain Tumor and Neuro-Oncology Center, Cleveland Clinic, Cleveland, OH, United States

**Keywords:** brain metastases (BM), stereotactic radiosurgery (SRS), radiation therapy (XRT), systemic therapy, review (article)

## Abstract

Brain metastases remain the most common neurologic complication of cancer. With improvement in surveillance and systemic therapy, patients with limited CNS disease are living longer after diagnosis, thus influencing the importance of optimal radiation treatment in order to maximize local control and minimize morbidity. In patients with a limited number of brain metastases, stereotactic radiosurgery is more recently seen as an appropriate sole modality for management with excellent local control. As newer systemic therapies emerge and with the advent of immunotherapies and targeted therapies for metastatic CNS disease, further research is needed in the optimal timing and sequencing of these modalities.

## Introduction

Up to 30% of cancer patients develop brain metastases during their lifetime making it the most common neurological complication of cancer (1, 2). The most common primary cancers that metastasize to the brain include lung cancer, breast cancer, kidney cancer and melanoma (3, 4). Incidence has increased due to more routine surveillance, detection of smaller lesions with MRI, as well as improved systemic therapies and thus improved length of survival. Given the available treatment options and strong proponents of various treatment options, optimal treatment has been controversial given the historically poor outcomes (5). While overall prognosis after development of brain metastases remains poor, a subset of patients can live several years after diagnosis, especially those with limited CNS disease (6). Given potential for long term survival, stereotactic radiosurgery (SRS), with or without whole-brain radiation (WBRT), has become an increasingly recognized standard of care in order to minimize morbidity. More recently, SRS alone has been supported as a sole modality for the management of1 to 4 brain metastases.

## Historical standards

The early randomized trials by Patchell et al. (7) answered initial questions about the best management strategy for single brain metastasis. In his initial study, patients with a single brain metastasis were randomized to surgery plus WBRT or biopsy plus WBRT which showed an overall survival (OS) benefit to surgical resection (40 vs. 15 weeks, *p* < 0.01) and local control improvement. Therefore, a subsequent study by Patchell et al. (8) was designed in which patients with a single brain metastasis had complete surgical resection and then randomized to WBRT or observation. Post-operative WBRT reduced intracranial failure from 70 to 18% (*p* < 0.001) and local recurrence (LR) from 46 to 10% (*p* < 0.001). Consequently, the optimal treatment of single brain metastasis was resection followed by WBRT. With the advent of SRS, future investigations focused on the addition of SRS to WBRT in order to improve local control (LC).

## WBRT + SRS

One of the earliest uses of SRS for brain metastases was as an adjunct to WBRT. At that time, the maximum number of brain metastases able to be treated was up to 3 or 4 due to technical limitations of the treatment machines. An initial study by Kondziolka et al. (9) randomized 27 patients with 2 to 4 brain metastases, all <2.5 cm, to WBRT vs. WBRT plus SRS boost. WBRT dose was 30 Gy in 12 fractions with an SRS boost of 16 Gy in a single fraction. Patients who received WBRT alone, had local failure rates of 100% vs. only 8% in patients who received SRS boost. Survival was 11 months in patients receiving SRS and 7.5 months in patients receiving WBRT alone (*p* = 0.22), which was expected given the small sample size that was underpowered to detect a survival difference. This data suggested that given poor LC rates with WBRT, SRS boost should be considered in patients with an otherwise reasonable survival expectation.

A subsequent larger randomized study (RTOG 95-08) (1) sought to further investigate the role of SRS boost. Three hundred, 33 patients with 1–3 brain metastases were randomized to WBRT vs. WBRT plus SRS boost. LC at 1 year improved from 71 to 82% with the addition of SRS (*p* = 0.01), though <50% of patients had adequate follow up imaging at 3 months. Overall, there was no difference in survival between the arms. In the subset of patients with single brain metastasis or recursive partition analysis (RPA) Class I, there was improved survival with SRS boost from 4.9 to 6.5 months (*p* = 0.39) and 9.6 to 11.6 months (*p* = 0.045), respectively. On secondary analysis (10), patients were classified by Graded Prognostic Assessment (GPA) score, a more modern prognostic scoring system compared to the RPA initially used. Patients with a high GPA (3.5–4) had improved survival regardless of number of brain metastases. This study further supported the observation that SRS boost improves LC and OS, particularly in patients with good performance status.

A Cochrane Database review updated in 2017 (11) synthesized available data regarding the benefit of SRS boost after WBRT. This review included three randomized trials which included a total of 358 patients. There was decreased local failure in the WBRT plus SRS group (HR 0.27 95% CI 0.14–0.52) as well as an improvement in performance status scores and decreased steroid use (RR 0.64 CI 0.42–0.97). There was no difference in OS in either group, though in participants with single brain metastasis had significantly longer median survival in the WBRT plus SRS group (*p* = 0.04).

## SRS alone

Subsequent data indicated there may be an association between WBRT and neurocognitive decline as well as an increased risk of dementia, though data was conflicting and some argued that progressive CNS disease caused more deleterious side effects than those related to WBRT (12–14). Thus, future studies focused on maximizing control, while further investigating effects of progressive brain metastases and treatment on neurocognition and quality of life. The debate surrounding the need for upfront WBRT in patients with a limited number of brain metastases was the subject of multiple future investigations. There have been four randomized trials investigating SRS alone vs. SRS plus WBRT (Table 1), which overall, have indicated that SRS alone allows for reduced effects on neurocognition, while still effectively managing brain metastases.

**Table 1:** Summary of SRS alone vs. SRS + WBRT.

	**Arm**	**1 year LC (%)**	**OS (months)**	**Clinical outcomes**
Aoyama et al. (15)	SRS SRS + WBRT	72.5 (*p* = 0.002) 88.7	8.0 (*p* = 0.42) 7.5	No difference in MMSE scores between groups
Chang et al. (16)	SRS SRS + WBRT	67 (*p* = 0.012) 100	15.2 (*p* = 0.003) 5.7	Decline in HVLT-R scores in SRS + WBRT arm
Kocher et al. (17)	SRS SRS + WBRT	69 (2y, *p* = 0.04) 81 (2y)	10.7 (*p* = 0.89) 10.9	Higher HRQOL scores in SRS alone arm (18)
Sahgal et al. (19)	SRS (* ≤* 50 y) SRS + WBRT (* ≤* 50 y) SRS (>50 y) SRS + WBRT (>50 y)	68 (crude rates) 89 74 88	13.6 8.2 10.1 8.6	Not reported
Brown et al. (20)	SRS SRS + WBRT	72.8 90.1	10.4 (*p* = 0.92) 7.4	Decline in immediate and delayed recall, verbal fluency, and executive functioning in WBRT arm

Aoyama et al. (15) published the first prospective study exploring this topic. In this phase III randomized control trial (RCT), 132 patients with 4 or less brain metastases <3 cm in size were randomized to SRS plus WBRT vs. SRS alone. The study was underpowered to detect an OS difference, and the primary endpoint was brain tumor recurrence. At 1 year, brain tumor recurrence decreased from 76 to 47% with the addition of WBRT (*p* < 0.001). WBRT also improved 1-year freedom from new brain metastases from 41.5% in SRS group to 64% (*p* = 0.003), and subsequently, there was more salvage treatment in the SRS alone group. There were no noted differences in toxicities between the groups. A subset of 28 patients had neurocognitive testing with Mini-Mental Status Examination (MMSE) at baseline and at least once at follow up. This group showed there was no difference after treatment between the two arms. Conflicting conclusions were drawn by various groups from this data, with the authors concluding that WBRT could be omitted safely, while others felt that WBRT improved LC and brain tumor recurrence and should be delivered routinely. In a secondary analysis of the data, published 9 years later, in the subset of patients with non-small cell lung cancer (NSCLC) with GPA score of 2.5–4, there was an improvement in OS from 10.6 to 16.7 months (*p* = 0.04) in patients receiving SRS plus WBRT (21). As expected, this group of patients had a lower rate of brain metastases recurrence (*p* < 0.01) which may contribute to improved OS. There was no improvement in survival for patients with lower GPA scores. This small sub-study of 47 patients is suggestive of benefit, though with small number of patients with 12 months follow up (*n* = 24), it may be considered hypothesis generating that maximal intracranial control is ideal for patients with potential for long survival.

More modern data have been acquired to further determine the neurocognitive impact of WBRT. Another phase III RCT compared SRS plus WBRT to SRS alone in patients with 1–3 brain metastasis and followed neurocognitive outcomes with the Hopkins Verbal Learning Test Revised (HVLT-R) (16). Fifty-eight patients were enrolled, and at interim analysis, there was a 96% probability that the SRS plus WBRT arm would show a decline in neurocognition, and the trial was ended early. As previously seen, there was a higher rate of CNS recurrence in SRS-only group compared to SRS plus WBRT, 73 vs. 27%, respectively (*p* = 0.0003). Median OS was surprisingly improved in SRS alone group at 15.2 vs. 5.7 months in the SRS plus WBRT group (*p* = 0.003). It was speculated that there was perhaps more surgical salvage and/or earlier start to systemic therapy in SRS alone group, or higher burden of systemic disease in those assigned to SRS plus WBRT. Given improved neurocognitive scores as well as potential for OS benefit, the authors concluded the SRS alone was preferred over SRS plus WBRT provided patients undergo close and careful follow up.

The European Organization for Research and Treatment of Cancer (EORTC) conducted a phase III trial in patients with 1–3 brain metastases who underwent SRS or surgery, then randomized patients to WBRT or observation (17). As expected, WBRT decreased the risk of intracranial relapse, however, there was no difference in OS between the groups. Interestingly, there was no difference in functional improvement between the two groups, indicating that while WBRT reduced the risk of recurrence, there was no clinical improvement in functional independence. Follow up publication by Soffietti et al. (18) focused on health-related quality of life (HRQOL) parameters in these patients. Patients in the observation arm had higher HRQOL scores in global health at 9 months (*p* = 0.148), as well as improved physical function and fatigue at 8 weeks, and cognitive functioning at 12 months compared to those in WBRT arm.

An individual patient-level meta-analysis of the above three studies was done to further characterize these findings. This showed that patients younger than 50 years old had improved survival with SRS alone when compared to SRS plus WBRT (10 vs. 8.2 months, *p* = 0.04). This patient group also had no difference in distant brain metastasis rate. It was concluded from this data set that the side effect profile of WBRT coupled with no improvement in distant brain metastasis rate may lead to the survival advantage seen in younger patients receiving SRS alone (19).

The most recent study investigating SRS vs. SRS plus WBRT was the results of the North Central Cancer Treatment Group (NCCTG) N0574 phase III study randomizing patients with 1–3 brain metastases to SRS vs. SRS plus WBRT (20). Two hundred eight patients were enrolled and the primary endpoint was neurocognitive function as defined as decline of >1 standard deviation from baseline in any of 7 cognitive domains at 3 months follow up. 91.7% of patients in the SRS plus WBRT arm had cognitive decline vs. 63.5% in SRS alone group (*p* < 0.001). Particular cognitive domains that were most affected by the addition of WBRT included immediate recall, delayed recall, and verbal fluency. In patients living 12 months or more, there was more frequent cognitive decline with the addition of WBRT, most notably in executive functioning (*p* = 0.05). However, there was improvement in 12 months intracranial control with addition of WBRT (84.6%) vs. SRS alone (50.5%). There was a numerical, though not statistically significant, improvement in median OS for SRS alone of 10.4 vs. 7.4 months (*p* = 0.92), though the study was not powered to detect OS differences. This larger study confirmed previous results (16), with a larger patient population, that in patients with 1–3 brain metastases, SRS alone may be preferred treatment modality.

From these four trials, we are able to glean several important points regarding the preferred treatment of patients with 1–4 brain metastases which were outlined by Arvold et al. (22). First, there is no negative impact on OS by eliminating WBRT in this patient population. Next, there is additive benefit in terms of LC with SRS plus WBRT, though SRS alone has similarly high rates of LC. Determining LC can be complicated by radiographic findings of pseudoprogression and radiation necrosis. Thirdly, when WBRT is withheld, there is increased rate of new distant brain metastases which leads to more frequent salvage treatment, and about a quarter of patients will ultimately require WBRT. Finally, the risk of neurocognitive decline is lower with SRS alone. Additionally, a Cochrane Database analysis of RCTs comparing of SRS or surgery alone vs. SRS or surgery plus whole brain further highlight the important data points (23). At 1 year, adding WBRT to SRS decreased relative risk of intracranial disease progression by 53%. However, there is no clear evidence of OS differences and subgroup analyses show similar OS regardless of therapy used, number of brain metastases as well as dose and sequence of WBRT.

With growing data as outlined above, ASTRO consensus guidelines were updated recommending against the routine use of WBRT in addition to SRS in patients with limited brain metastases. In addition, multiple other groups through editorials as well as groups such as The National Comprehensive Cancer Network (24), Deutsche Gesellschaft fur Radioonkologie (25), and International Stereotactic Radiosurgery Society (ISRS) (26) have voiced that SRS alone is favored in patients with limited brain metastasis burden and WBRT to be reserved for salvage options (27). Further studies have begun investigating the utility of SRS alone in >4 brain metastases. Yamamoto et al. reported their prospective observational study of SRS alone for treatment of 5–10 brain metastases compared to treated of two to four brain metastases (28). They found that overall survival was similar between patients with 2–4 metastases as compared to 5–10 metastases with no difference in acute toxicities. Future study is necessary to optimize appropriate settings for SRS alone.

## Optimal timing of SRS and systemic therapy

The typical approach for management of systemic disease with brain metastases is treatment of CNS disease first, followed by initiation of systemic therapy. A recent randomized trial out of Korea, specifically evaluated timing of SRS relative to the start of chemotherapy in patients with limited number of asymptomatic brain metastases (29). Patients with NSCLC were randomized to upfront SRS prior to chemotherapy initiation vs. initiation of chemotherapy without treatment of CNS disease. Median OS was equivalent between the groups, though there was a trend toward longer CNS progression free survival, lower symptomatic brain progression rate and lower CNS salvage rates in the upfront SRS group. It appears from this data, that upfront SRS may be preferable, though in cases that urgent chemotherapy is needed, delaying CNS treatment is likely safe.

New emerging data suggests that systemic therapy may be safely given concurrently with SRS. In retrospective studies, there does not appear to be an association between timing of systemic therapy and increased rates of myelosuppression. A retrospective review from Johns Hopkins showed that in patients receiving concurrent systemic therapy with SRS, only 4% of patients developed grade 3 or 4 neurotoxicity (30). There was an association between higher grade of neurotoxicity with concurrent use of immune therapy as well as lower use of steroids with concurrent targeted therapy. There was no difference in rates of radiation necrosis, grade of neurotoxicity, or steroid use based on timing of systemic therapies. Interestingly, in newly diagnosed cancer patients found to have brain metastases, treatment with concurrent systemic therapy and SRS had improved survival compared to SRS alone (41.6 vs. 21.5 months, *p* < 0.05). In a larger retrospective review of 1,650 patients with 27% of patients receiving concurrent systemic therapy, similar results were found. In patients who received SRS plus WBRT, there was a higher rate of radiation necrosis, compared to SRS alone when patients received concurrent vascular endothelial growth factor receptor tyrosine kinase inhibitors (TKIs; 14.3 vs. 6.6%, *p* = 0.04) or epidermal growth factor receptor TKIs (15.6 vs. 6% *p* = 0.04). There was no association between other systemic therapies, including hormonal therapy, cytotoxic chemotherapy or other targeted agents, and risk of radiation necrosis when given concurrently with SRS (31). Similar results were seen in secondary analysis of patients enrolled on RTOG 0320 and concurrent use of temozolomide or erlotinib with concurrent SRS or SRS plus WBRT. This analysis showed that patients had more toxicity and worse survival when receiving either systemic agent in combination with WBRT plus SRS vs. WBRT or SRS alone (32).

In the era of new targeted therapies, the indications and timing of SRS is not always clear, and in some cases radiation may be deferred for immediate targeted therapy start. A recent multi-institutional retrospective review evaluated 351 patients with EGFR-mutant NSCLC with new brain metastases who were TKI naïve (33). Patients were treated with SRS or WBRT followed by TKI therapy or TKI therapy alone with radiation reserved at time of progression. Outcomes showed that delaying radiation, WBRT or SRS alone, is associated with significantly worse OS in this patient population. Patients treated with SRS followed by TKI had the longest median OS at 46 months, compared to 30 months with WBRT + TKI and 25 months with TKI alone (*P* < 0.001 for each group). Further randomized data is needed to better define the optimal timing and sequencing of radiation and systemic therapy, particularly in the setting of new targeted therapies.

## Conclusion

Historically, WBRT was used in conjunction with SRS in order to improve intracranial control, with major disadvantage being neurocognitive decline with the addition of WBRT. In the era of improved surveillance with MRI imaging, better systemic therapy, and improved patient survival, goals have transformed to limit late toxicity, particularly in favorable patient populations with limited CNS disease. Multiple studies have shown that SRS alone for 1–4 brain metastases has acceptable local control with reduced neurocognitive decline as compared to WBRT, and thus, is the favored treatment modality in this patient population (15–17, 20, 27). SRS alone may be appropriate for patients with >4 brain metastases, though further study is necessary to clarify optimal patient selection.

## Author contributions

SS and SC: development, writing, and editing; JS, EM, and JY: writing and editing.

### Conflict of interest statement

The authors declare that the research was conducted in the absence of any commercial or financial relationships that could be construed as a potential conflict of interest. The handling editor and reviewer AS declared their involvement as co-editors in the Research Topic, and confirm the absence of any other collaboration.
